# Investigating the Electrochemical Performance of MnFe_2_O_4_@xC Nanocomposites as Anode Materials for Sodium-Ion Batteries

**DOI:** 10.3390/molecules29163912

**Published:** 2024-08-19

**Authors:** Shi-Wei Liu, Bai-Tong Niu, Bi-Li Lin, Yuan-Ting Lin, Xiao-Ping Chen, Hong-Xu Guo, Yan-Xin Chen, Xiu-Mei Lin

**Affiliations:** 1 College of Chemistry, Chemical Engineering and Environment, Minnan Normal University, Zhangzhou 363000, Chinabaitongniu@163.com (B.-T.N.); 15359025346@163.com (Y.-T.L.); xiaopingchen@mnnu.edu.cn (X.-P.C.); guohx@mnnu.edu.cn (H.-X.G.); 2 State Key Laboratory of Structural Chemistry, Fujian Institute of Research on the Structure of Matter, Chinese Academy of Sciences, Fuzhou 350002, China; yanxinchen@fjirsm.ac.cn

**Keywords:** sodium-ion batteries, transition metal oxides (TMOs), MnFe_2_O_4_, carbon coating, electrochemical performance

## Abstract

Transition metal oxides (TMOs) are important anode materials in sodium-ion batteries (SIBs) due to their high theoretical capacities, abundant resources, and cost-effectiveness. However, issues such as the low conductivity and large volume variation of TMO bulk materials during the cycling process result in poor electrochemical performance. Nanosizing and compositing with carbon materials are two effective strategies to overcome these issues. In this study, spherical MnFe_2_O_4_@xC nanocomposites composed of MnFe_2_O_4_ inner cores and tunable carbon shell thicknesses were successfully prepared and utilized as anode materials for SIBs. It was found that the property of the carbon shell plays a crucial role in tuning the electrochemical performance of MnFe_2_O_4_@xC nanocomposites and an appropriate carbon shell thickness (content) leads to the optimal battery performance. Thus, compared to MnFe_2_O_4_@1C and MnFe_2_O_4_@8C, MnFe_2_O_4_@4C nanocomposite exhibits optimal electrochemical performance by releasing a reversible specific capacity of around 308 mAh·g^−1^ at 0.1 A·g^−1^ with 93% capacity retention after 100 cycles, 250 mAh·g^−1^ at 1.0 A g^−1^ with 73% capacity retention after 300 cycles in a half cell, and around 111 mAh·g^−1^ at 1.0 C when coupled with a Na_3_V_2_(PO_4_)_3_ (NVP) cathode in a full SIB cell.

## 1. Introduction

With the growing depletion of traditional fossil fuels and the resultant environmental challenges, new energy sources with robust advancement have emerged as a pivotal concern for the sustainable progress of human society. Currently, intermittent renewable energy sources such as solar energy, hydropower, and wind energy are affected by natural conditions, such as weather changes and climate anomalies, which affect the energy supply and cannot guarantee stability [1,2]. Consequently, the development of energy storage technology has become increasingly vital in addressing the stability issues associated with energy supply [3]. Among numerous secondary energy systems, sodium-ion batteries (SIBs) have been widely studied as an alternative to lithium-ion batteries (LIBs) due to their abundant and homogeneous sodium resources, low cost, and high safety ratings [4]. To date, a plethora of conversion-based materials, such as transition metal oxides (TMOs)/sulfides/phosphides/selenides [5,6,7,8], carbon-based materials [9], alloying materials [10], and organic compounds [11], have demonstrated fantastic electrochemical activity as anode materials for SIBs.

Among all these anode materials, TMOs have piqued the interest of researchers because of their abundant redox-active properties, low cost, high energy density, ease of preparation, and environmental benignity [12]. TMOs such as MnFe_2_O_4_ [13], CuMn_2_O_4_ [14], FeCo_2_O_4_ [15], FeV_2_O_4_ [16], ZnFe_2_O_4_ [17], ZnCo_2_O_4_ [18], and ZnMn_2_O_4_ [19] have been explored as anode materials for SIBs. However, the intrinsically poor electric conductivity and uncontrollable and inevitable volume changes in bulk TMO electrode materials during the cycling process lead to poor electrochemical performance [20,21], seriously hindering the practical application of TMO-based electrode materials. Composite formation and nanosizing of carbon materials are two effective strategies to solve the above-mentioned problems. Nanosizing of the carbon composite can improve the effective contact area of the TMO electrode with electrolytes, mitigating irreversible damage to the composition of the electrode caused by volume changes during the charge–discharge cycle, shortening ion diffusion distance and, thus, enhancing the specific capacity, rate capability, and long-term cycle reversibility of batteries. Composite formation with carbon materials [17,18,19,21,22,23,24], especially the coating of a carbon shell onto TMO nanoparticles, enhances the electric conductivity, prevents the agglomeration of nanoparticles, and strengthens the mechanical integrity of electrode materials, thereby improving the overall electrochemical performance of batteries.

In recent years, nanocomposite materials composed of TMO cores and carbon shells have been widely studied to improve the conductivity and mechanical properties of TMOs@C to improve their electrochemical performance as anode materials for SIBs [17,23,24]. Studies of alloying-conversion-type ZnFe_2_O_4_@xC SIB anode materials have shown that enhanced electrochemical performance can be achieved by increasing the carbon shell thickness of the nanocomposites [17]. In this work, we synthesized conversion-type MnFe_2_O_4_ spherical nanoparticles using a hydrothermal method and coated them with different thicknesses of carbon shell by tuning the mass of polyvinylidene fluoride (PVDF) for pyrolysis to prepare core–shell-structure MnFe_2_O_4_@xC nanocomposites to study the influence of the thickness of the carbon shell on the electrochemical performance of conversion-type SIB anode materials. MnFe_2_O_4_@xC nanocomposites with carbon shell contents of 3.28%, 12.89%, and 26.30% (mass ratio) are denoted as MnFe_2_O_4_@1C, MnFe_2_O_4_@4C, and MnFe_2_O_4_@8C, respectively. The sodium-ion storage performance of these nanocomposites was explored in a half cell. At a specific current of 0.1 A·g^−1^, MnFe_2_O_4_@4C (308 mAh·g^−1^) and MnFe_2_O_4_@1C (295 mAh·g^−1^) nanocomposites show higher specific capacities than MnFe_2_O_4_@8C (195 mAh·g^−1^), while MnFe_2_O_4_@4C and MnFe_2_O_4_@8C show better cycling stabilities than MnFe_2_O_4_@1C in a comparison of 93%, 99%, and 71% capacity retentions after 100 cycles. A similar phenomenon can be observed when the specific current is 1.0 A g^−1^. MnFe_2_O_4_@4C and MnFe_2_O_4_@8C show better rate capabilities than MnFe_2_O_4_@1C. With 40 folds of specific current increment (from 0.05 to 2.0 A g^−1^), the capacity retentions of MnFe_2_O_4_@4C, MnFe_2_O_4_@8C, and MnFe_2_O_4_@1C are 65%, 63%, and 44%, respectively.

Transmission electron microscopy (TEM), electrochemical impedance spectroscopy (EIS), and kinetic analysis show that the carbon shell plays a crucial role in tuning the electrochemical performance of MnFe_2_O_4_@xC nanocomposites. The electric conductivity and the connectivity among particles of MnFe_2_O_4_@xC nanocomposites increase with the thickness of the carbon shell (content), which leads to enhanced rate capability and cycling stability. Therefore, MnFe_2_O_4_@4C and MnFe_2_O_4_@8C show superior rate capability and cycling stability relative to MnFe_2_O_4_@1C. Nevertheless, further increasing the thickness of the carbon shell results in a smaller ratio of active MnFe_2_O_4_ core material (the specific capacity was calculated based on the mass of the whole MnFe_2_O_4_@xC nanocomposites), leading to a decreased specific capacity, which explains the lower specific capacity of MnFe_2_O_4_@8C than that of MnFe_2_O_4_@1C and MnFe_2_O_4_@4C. In summary, MnFe_2_O_4_@4C with an appropriate carbon shell thickness (content) leads to optimal battery performance with a reversible specific capacity of around 308 mAh·g^−1^ at 0.1 A·g^−1^ with 93% capacity retention after 100 cycles and 250 mAh·g^−1^ at 1.0 A g^−1^ with 73% capacity retention after 300 cycles in a half cell. Finally, the full SIB cell performance of a MnFe_2_O_4_@4C anode coupled with a Na_3_V_2_(PO_4_)_3_ (NVP) cathode was also tested, showing a specific capacity of around 111 mAh·g^−1^ at 1.0 C. Our findings show the importance of tuning the thickness of the carbon shell to achieve superior battery performance of TMO SIB anodes.

## 2. Results and Discussion

### 2.1. Structure and Morphology Analysis

**Figure 1:**
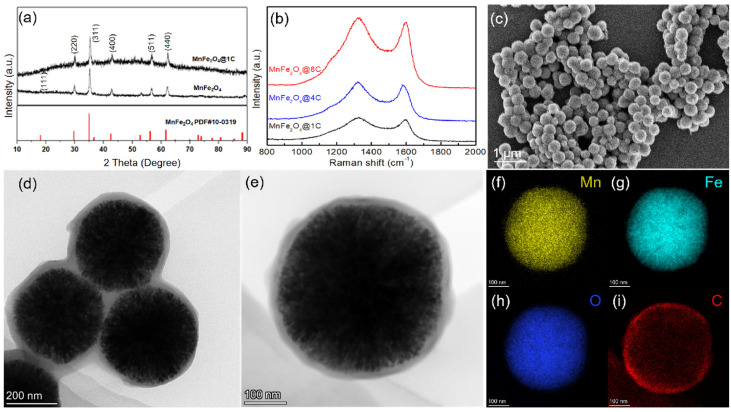
(**a**) Powder XRD patterns of MnFe_2_O_4_ and MnFe_2_O_4_@1C. (**b**) Raman spectra of MnFe_2_O_4_@1C, MnFe_2_O_4_@4C, and MnFe_2_O_4_@8C nanocomposites. (**c**) SEM images of MnFe_2_O_4_@1C nanocomposite. (**d**,**e**) TEM images of MnFe_2_O_4_@1C nanocomposite. (**f**–**i**) Elemental mapping images of MnFe_2_O_4_@1C.

The crystallinity and phase purity of the MnFe_2_O_4_, MnFe_2_O_4_@1C, MnFe_2_O_4_@4C, and MnFe_2_O_4_@8C samples were characterized using an X-ray diffraction (XRD) technique, the results of which are shown in Figure 1a and Figure S2b, indicating the cubic property with the Fd-3m space group of both materials [25,26]. The main peaks at 2*θ* (in degree) values of 18.1°, 29.7°, 35.0°, 42.5°, 56.2°, and 61.7° of the XRD spectra correspond to (1 1 1), (2 2 0), (3 1 1), (4 0 0), (5 1 1), and (4 4 0) crystal planes, respectively [27]. They are essentially consistent with the MnFe_2_O_4_ standard database (PDF#10-0319). In addition, the peak positions of the diffraction peaks of the MnFe_2_O_4_ and MnFe_2_O_4_@1C samples are similar to each other, indicating that the carbon coating did not change the crystallinity and phase of MnFe_2_O_4_. The Raman spectra of the MnFe_2_O_4_@1C, MnFe_2_O_4_@4C, and MnFe_2_O_4_@8C nanocomposites (Figure 1b) show two prominent peaks at approximately 1330 cm^−1^ and 1590 cm^−1^, which correspond to the D and G bands, respectively, indicating the complete calcination of PVDF into carbon.

**Figure 2:**
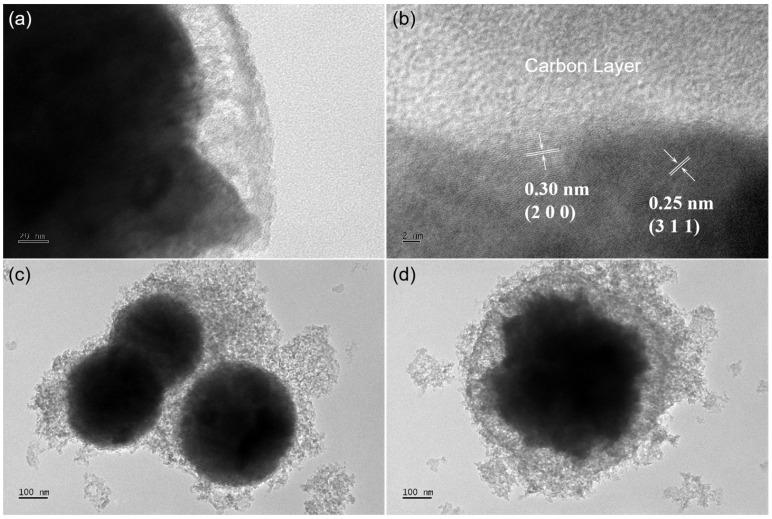
(**a**,**b**) HRTEM images of MnFe_2_O_4_@4C. (**c**,**d**) TEM images of MnFe_2_O_4_@8C.

Figure 1c shows a SEM image of MnFe_2_O_4_@1C nanocomposite, and the results reveal that the synthesized material primarily consists of spherical nanoparticles with a diameter of approximately 400 nm. The SEM images of MnFe_2_O_4_ also show similar spherical nanoparticles (Figure S1). The TEM images of MnFe_2_O_4_@1C in Figure 1d,e show that the MnFe_2_O_4_ inner core is surrounded by a thin carbon coating layer of approximately 10 nm. The element mapping images of the MnFe_2_O_4_@1C nanocomposite are depicted in Figure 1f–i. The evident distribution of Mn, Fe, O, and C elements in the MnFe_2_O_4_@1C nanocomposites further indicates the spherical core–shell nanostructure of the MnFe_2_O_4_@1C nanocomposite, with the C element shell loaded on the surface of the MnFe_2_O_4_ inner core. The HRTEM image of MnFe_2_O_4_@4C in Figure 2a illustrates that the MnFe_2_O_4_ core is surrounded by a carbon coating of 20 to 30 nm. Furthermore, crystal plane stripes with lattice spacings of 0.25 and 0.30 nm are depicted in Figure 2b, which can be attributed to the (3 1 1) and (2 2 0) crystal planes of MnFe_2_O_4_ core, respectively [28]. TEM images of the MnFe_2_O_4_@8C nanocomposite in Figure 2c,d show that the thickness of the carbon shell is approximately 100 nm. As can be observed from the TEM images, the thickness of the carbon shell increases following a sequence from MnFe_2_O_4_@1C and MnFe_2_O_4_@4C to MnFe_2_O_4_@8C, which enhances the connectivity among nanoparticles.

The thermal decomposition process of MnFe_2_O_4_@xC was measured in an Ar atmosphere and is shown in Figure S2b. The three MnFe_2_O_4_@xC samples exhibit similar TGA curves, with weight loss primarily occurring at >450 °C, which can be attributed to the loss of carbon coating in the outer layer of the composite materials. The residual mass at the end of the TG curve is attributed to the residue of MnFe_2_O_4_, demonstrating the thermal stability of MnFe_2_O_4_ core material, even at 1000 °C. Therefore, the carbon content in the three nanocomposite materials is 3.28%, 12.89%, and 26.30%, as denoted by MnFe_2_O_4_@1C, MnFe_2_O_4_@4C, and MnFe_2_O_4_@8C, respectively.

### 2.2. Sodium-Ion Storage Performance of the MnFe_2_O_4_@xC Nanocomposites

**Figure 3:**
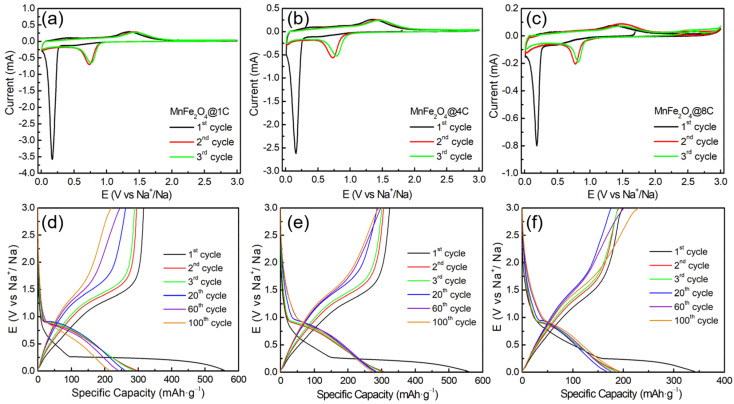
Initial three CV curves of (**a**) MnFe_2_O_4_@1C, (**b**) MnFe_2_O_4_@4C, and (**c**) MnFe_2_O_4_@8C. Galvanostatic discharge–charge profiles of (**d**) MnFe_2_O_4_@1C, (**e**) MnFe_2_O_4_@4C, and (**f**) MnFe_2_O_4_@8C at 0.1 A·g^−1^ in a Na half cell (0.01–3.0 V).

**Figure 4:**
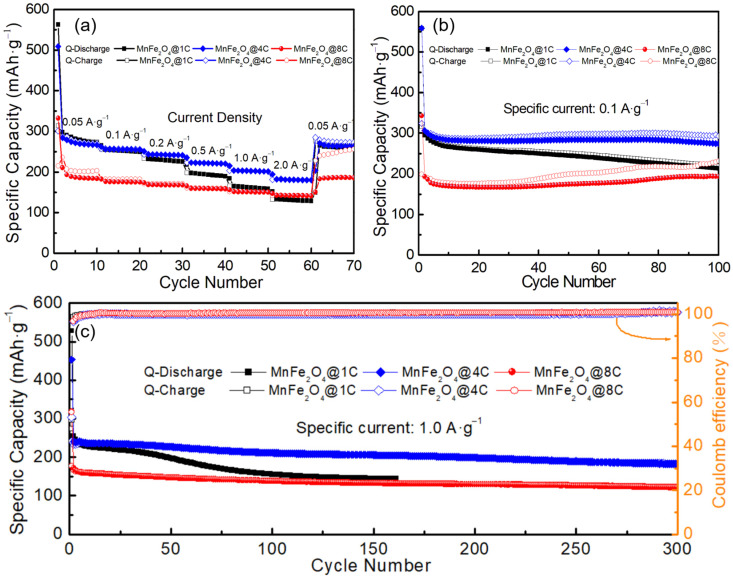
Rate capability (**a**) and cycling stability of MnFe_2_O_4_@1C, MnFe_2_O_4_O@4C, and MnFe_2_O_4_@8C at specific currents of (**b**) 0.1 A·g^−1^ and (**c**) 1.0 A·g^−1^ in a Na half–cell.

The Na^+^ storage performance of the three MnFe_2_O_4_@xC nanocomposites was evaluated, as presented in Figure 3 and Figure 4. As shown in Figure 3a–c, the CV curves of the three materials show similar features. During the first cathodic scan, a sharp cathodic peak at ~0.17 V is observed before disappearing in the subsequent cycle, which is mainly attributed to irreversible electrochemical processes such as the transformation of MnFe_2_O_4_ to metallic Mn and Fe [29] and the formation of SEI, as represented in Equation (1). The anodic peak around 1.34 V is assigned to the oxidation of Mn and Fe, leading to the formation of MnO and Fe_2_O_3_, respectively, as indicated in Equation (2).

MnFe_2_O_4_ + 8Na^+^ + 8e^−^ → Mn + 2Fe + 4Na_2_O, (1)

Mn + 2Fe + 4Na_2_O → MnO + Fe_2_O_3_ + 8Na^+^ + 8e^−^, (2)

In the second and third cycles, a cathodic peak shows up at ~0.76 V, indicating the reversible reduction reactions of MnO to Mn and Fe_2_O_3_ to Fe (Equations (3) and (4)) [30,31]. From the second cycle onwards, it is noted that the majority of the CV curves overlap, indicating the favorable reversibility of the electrochemical reactions, as described in Equations (5) and (6) [13,32,33,34].

MnO + 2Na^+^ + 2e^−^ → Mn + Na_2_O, (3)

Fe_2_O_3_ + 6Na^+^ + 6e^−^ → 2Fe + 3Na_2_O, (4)

Overall: MnO + Fe_2_O_3_ + 8Na^+^ + 8e^−^ ↔ Mn + 2Fe + 4Na_2_O, (5)

*x*C+ Na^+^ +e^−^ ↔ NaC*_x_*, (6)

Figure 3d–f show the galvanostatic discharge–charge profiles of MnFe_2_O_4_@1C, MnFe_2_O_4_@4C, and MnFe_2_O_4_@8C at 0.1 A·g^−1^ in Na half cells. In the first discharge–charge profiles, all samples exhibit irreversible capacity loss, which is attributed to the formation of an SEI layer and the irreversible transformation of MnFe_2_O_4_. The first discharge capacities of MnFe_2_O_4_@1C, MnFe_2_O_4_@4C, and MnFe_2_O_4_@8C are 558, 559, and 343 mAh·g^−1^, respectively. Starting from the second cycle, the discharge–charge capacities of MnFe_2_O_4_@1C, MnFe_2_O_4_@4C, and MnFe_2_O_4_@8C show good reversibility, with values of 295/295, 308/307, and 195/194 mAh·g^−1^, respectively. MnFe_2_O_4_@4C shows a notable improvement in specific capacity compared to MnFe_2_O_4_@1C and MnFe_2_O_4_@8C. With increasing cycle number (1st to 100th cycle), the capacity of MnFe_2_O_4_@1C decays, while that of MnFe_2_O_4_@4C and MnFe_2_O_4_@8C remains constant.

The rate capabilities of MnFe_2_O_4_@1C, MnFe_2_O_4_@4C, and MnFe_2_O_4_@8C in Na half cells were tested, as shown in Figure 4a. With the specific current increasing from 0.05 A g^−1^ through 0.1, 0.2, 0.5, and 1.0 to 2.0 A g^−1^ (40 folds), the specific capacity retentions of MnFe_2_O_4_@1C, MnFe_2_O_4_@4C, and MnFe_2_O_4_@8C are 44%, 65%, and 63% (from 295, 286, and 210 mAh·g^−1^ to 131, 182, and 132 mAh·g^−1^, respectively). MnFe_2_O_4_@4C and MnFe_2_O_4_@8C show better rate capabilities than MnFe_2_O_4_@1C. Further research was conducted on the cycling stability of MnFe_2_O_4_@xC electrodes in Na half cells. Figure 4b depicts the long-term cycling performance of MnFe_2_O_4_@1C, MnFe_2_O_4_@4C, and MnFe_2_O_4_@8C electrodes at 0.1 A·g^−1^. The results indicate that after 100 cycles, the three electrodes achieved discharge capacities of 220, 274, and 193 mAh·g^−1^ from the initial discharge capacities of 308, 295, and 195 mAh·g^−1^, with capacity retentions of 71%, 93%, and 99%, respectively. At a specific current of 1.0 A·g^−1^ (Figure 4c), the initial capacities of the MnFe_2_O_4_@4C and MnFe_2_O_4_@8C electrodes are 250 and 166 mAh·g^−1^, decaying to 183 and 129 mAh·g^−1^ after 300 cycles, indicating capacity retentions of around 73% and 78%, respectively. The MnFe_2_O_4_@1C electrode shows inferior cycling stability relative to the other two electrodes. In summary, MnFe_2_O_4_@1C and MnFe_2_O_4_@4C show higher specific capacity than MnFe_2_O_4_@8C, while MnFe_2_O_4_@4C and MnFe_2_O_4_@8C show superior rate capability and cycling stability relative to MnFe_2_O_4_@1C. Comprehensively considering the specific capacity, rate capability, and cycling stability, MnFe_2_O_4_@4C achieves optimal electrochemical battery performance. A comparison of the SIB anode performance of the MnFe_2_O_4_@4C nanocomposite with other TMOs is shown in Table 1, revealing the superior electrochemical performance of our material.

### 2.3. Exploring the Origin of the Improvement in the Sodium-Ion Storage of the MnFe_2_O_4_@xC Nanocomposites

**Figure 5:**
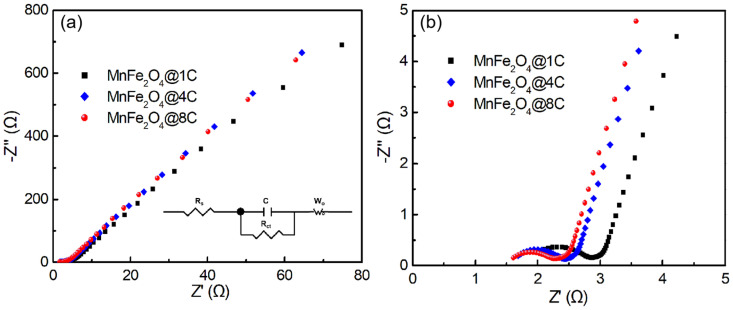
(**a**) AC impedance measurements of MnFe_2_O_4_@1C, MnFe_2_O_4_@4C, and MnFe_2_O_4_@8C nanocomposite electrodes. The insert in (**a**) is the equivalent circuit diagram. (**b**) The zoomed−in area of the plots in the high-frequency region of (**a**).

Figure 5a illustrates the results of an EIS test on the three MnFe_2_O_4_@xC nanocomposites. The Nyquist curves of the three materials exhibit similar trends, comprising a semicircle in the high-frequency region and an inclined straight line in the low-frequency region (Figure 5b). The cell resistances (*R*_s_, calculated from the intercept of the *X*-axis) for the MnFe_2_O_4_@1C, MnFe_2_O_4_@4C, and MnFe_2_O_4_@8C Na half cells are 1.86, 1.59, and 1.54 Ω, respectively. The diameters of the semicircles represent the charge transfer resistance (*R*_ct_) coming from the electrode material/electrolyte interface reaction, which are 0.84, 0.70, and 0.62 Ω for the MnFe_2_O_4_@1C, MnFe_2_O_4_@4C, and MnFe_2_O_4_@8C nanocomposite electrodes, respectively. The *R*_ct_ increases gradually with the increase in the thickness (content) of the carbon shell in the core–shell nanostructure due to the good electric conductivity of the carbon material, which improves the electric conductivity of the MnFe_2_O_4_@xC core–shell nanocomposites as a whole. Good electric conductivity leads to good rate capability. Moreover, as observed in Figure 1 and Figure 2, the particle connectivity of MnFe_2_O_4_@xC nanocomposites increases with the increase in carbon shell thickness (content), which leads to enhanced rate capability and cycling stability. Therefore, MnFe_2_O_4_@4C and MnFe_2_O_4_@8C show superior rate capability and cycling stability relative to MnFe_2_O_4_@1C. Nevertheless, a thick carbon shell results in a small ratio of active MnFe_2_O_4_ core material (the specific capacity was calculated based on the mass of the whole MnFe_2_O_4_@4C nanocomposite), leading to a decreased specific capacity, which explains the lower specific capacity of MnFe_2_O_4_@8C relative to MnFe_2_O_4_@1C and MnFe_2_O_4_@4C. In summary, the use of MnFe_2_O_4_@4C with an appropriate carbon shell thickness (content) leads to optimal battery performance.

**Figure 6:**
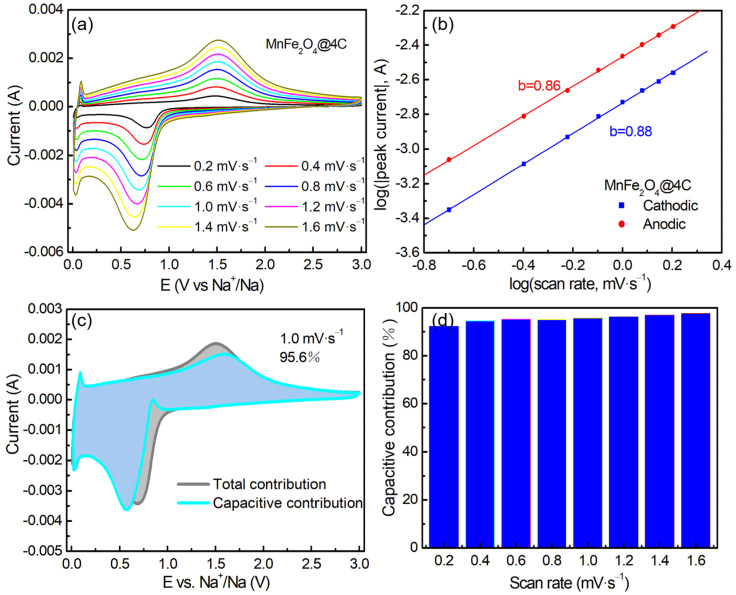
(**a**) CVs at various scan rates. (**b**) Calculated b values. (**c**) Capacitive contribution at a scan rate of 1.0 mV·s^−1^. (**d**) The ratio of capacitive contribution of the MnFe_2_O_4_@4C anode at various scan rates.

To systematically investigate the electrochemical reaction kinetics of MnFe_2_O_4_@4C as an anodic electrode material for Na half cells, the corresponding CV curves were tested, with the scan rate increasing from 0.2 to 1.6 mV·s^−1^ (Figure 6a). The CV curves of MnFe_2_O_4_@4C at different scanning rates exhibit similar shapes, indicating that the material has low polarization and good electrochemical reaction kinetics. Under different scanning rates (*v*), the capacitance behavior or diffusion behavior of the electrode is obtained by deducing the current (*i*) according to the following formula (Equations (7) and (8)) [43,44,45]:

*i* = a*v*^b^, or (7)

log *i* = b log *v* + log a, (8)

where a is a constant and b represents the capacitance ratio, which can be determined by the slope of the logarithmic curve (log (*i*) vs. log (*v*)). A value of b = 0.5 indicates a diffusion-controlled behavior, while a value of b = 1.0 signifies a pseudocapacitance-controlled contribution. In Figure 6b, the slope of the redox process is depicted, with the b values of cathodic and anodic currents being 0.86 and 0.88, respectively. This suggests that the sodium storage behavior in MnFe_2_O_4_@4C is primarily influenced by pseudocapacitance. Additionally, we consider Equation (9) [46],

*i* (V) = k_1_*v* + k_2_*v*^1/2^, (9)

where the current (*i*) at a fixed potential (V) in CV can be divided into two parts, namely capacitance behavior (k_1_*v*) and diffusion control (k_2_*v*^1/2^). The capacitive contribution of the MnFe_2_O_4_@4C half cell at 1.0 mV·s^−1^ is shown in Figure 6c, which is measured to be 95.6% of the total contribution. The respective contributions of capacitance behavior and diffusion control at different scan rates are depicted in Figure 6d. The contribution of capacitance behavior increases as the scan rate rises, indicating that the pseudocapacitance process in the MnFe_2_O_4_@4C Na half cell becomes more prominent at higher scan rates, demonstrating faster kinetics. At a scanning rate of 1.6 mV·s^−1^, a capacitance contribution of 97.5% is achieved. Even at a low rate of 0.2 mV·s^−1^, MnFe_2_O_4_@4C still significantly contributes to the capacitance behavior (92.2%). MnFe_2_O_4_@1C and MnFe_2_O_4_@8C show similar electrochemical reaction kinetics to those of MnFe_2_O_4_@4C, with the b values of cathodic and anodic currents of the MnFe_2_O_4_@1C electrode of 0.85 and 0.84, respectively, while those of the MnFe_2_O_4_@8C electrode are 0.86 and 0.90, respectively (Figure S3).

### 2.4. Full-Cell Performance

**Figure 7:**
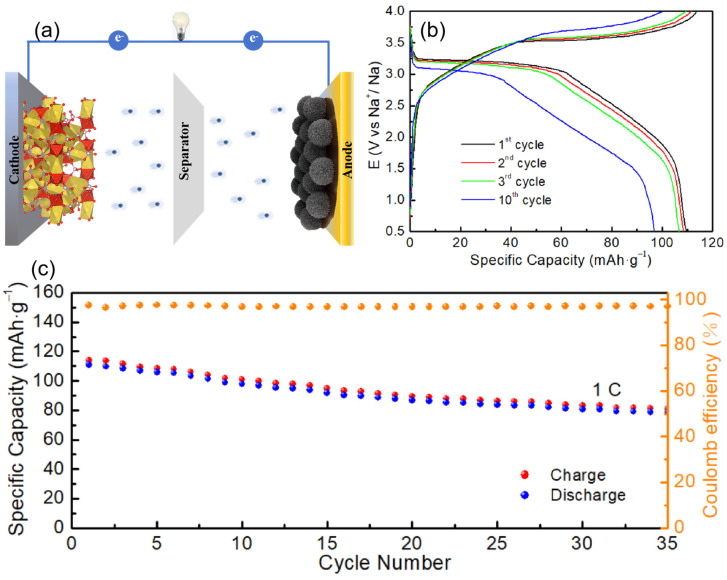
NVP//MnFe_2_O_4_@4C full cell: (**a**) illustrative diagram; (**b**) voltage-versus-capacity profiles at 1.0 C during cycling at different cycle numbers; (**c**) cycling performance at 1.0 C.

To further assess the practical usability of MnFe_2_O_4_@4C in SIBs, full-cell configurations were assembled, with Na_3_V_2_(PO_4_)_3_ (NVP) serving as the cathode and MnFe_2_O_4_@4C as the anode (Figure 7a). The charge–discharge profiles of the NVP//MnFe_2_O_4_@4C full SIB at 1 C-rate (1 C-rate = 120 mA·g^−1^) within the voltage range of 0.5–4.0 V are presented in Figure 7b. In the initial cycle, NVP//MnFe_2_O_4_@4C SIB demonstrates a specific capacity of 111 mAh·g^−1^ at 1C. As illustrated in Figure 7c, the full cell exhibits favorable electrochemical performance, with a stable and reversible capacity of 78.5 mAh·g^−1^ over 35 cycles, and maintains a Coulombic efficiency of over 97% throughout the cycling process.

## 3. Materials and Methods

### 3.1. Synthesis of the MnFe_2_O_4_ Spherical Nanoparticles

The MnFe_2_O_4_ spherical nanoparticles were synthesized using a hydrothermal method based on the methods reported in [47], with some modifications. Initially, MnCl_2_·4H_2_O (0.396 g, 2 mmol) and FeCl_3_·6H_2_O (1.081 g, 4 mmol) were added to 80 mL of ethylene glycol and stirred to form a solution. Subsequently, NaAc (7.2 g) and polyethylene glycol (2.0 g, M = 20,000) were added. After stirring for 12 h, the resulting homogeneous solution was transferred into a 100 mL autoclave with a Teflon^®^ lining and heated at 200 °C for 10 h. After being cooled to room temperature, the resulting brown precipitate was collected via centrifugation at 10,000 rpm, washed several times with ultrapure water and absolute ethanol, and dried at 60 °C for 6 h.

### 3.2. Synthesis of the MnFe_2_O_4_@xC Spherical Nanocomposites

The carbon shell was coated onto the MnFe_2_O_4_ core to form MnFe_2_O_4_@xC core–shell nanocomposites by pyrolysis of PVDF [48,49]. In general, 16.0 mg of PVDF was dissolved in a beaker containing 280.0 mg of N-methylpyrrolidone (NMP) with a PVDF mass ratio of 6%. Then, 100 mg of MnFe_2_O_4_ spherical nanoparticles was added to the above solution and stirred for 24 h. The mixture was then dried at 120 °C for 24 h and moved to a combustion boat, where it was crystallized and carbonized in an Argon flow at 700 °C for 2 h at a ramp rate of 2 °C/min. The product obtained after natural cooling is denoted as MnFe_2_O_4_@1C. Similarly, after increasing the mass of PVDF to 64.0 and 128.0 mg for pyrolysis, the obtained products were referred to as MnFe_2_O_4_@4C and MnFe_2_O_4_@8C, respectively.

Comprehensive details regarding material characterization, electrochemical measurements, and kinetics analysis can be found in the Supplementary Materials.

## 4. Conclusions

In this work, MnFe_2_O_4_@xC nanocomposites were successfully synthesized by a hydrothermal method with MnFe_2_O_4_ inner cores, and the thicknesses of the carbon shells were tuned by pyrolysis of PVDF. The electrochemical performance of MnFe_2_O_4_@xC nanocomposites as anode materials for SIBs was evaluated. Compared to MnFe_2_O_4_@1C and MnFe_2_O_4_@8C, MnFe_2_O_4_@4C nanocomposite achieves optimal electrochemical performance. It releases a reversible specific capacity of around 308 mAh·g^−1^ at 0.1 A·g^−1^ and 73% capacity retention after 100 cycles and 250 mAh·g^−1^ at 1.0 A g^−1^ with 73% capacity retention after 300 cycles in a half cell and of around 111 mAh·g^−1^ at 1.0 C coupled with a Na_3_V_2_(PO_4_)_3_ (NVP) cathode in a full SIB cell. TEM, EIS, and kinetic analysis show that the optimal electrochemical performance of MnFe_2_O_4_@4C is due to its appropriate carbon shell thickness (content) of 20–30 nm (12.89% mass ratio), which leads to the optimal electric conductivity, particle connectivity, and kinetics of the electrode material, as well as enhanced specific capacity, rate capability, and cycling stability.

## Figures and Tables

**Table 1:** Comparison of the SIB performance of the MnFe_2_O_4_@4C nanocomposite with other TMOs.

Materials	Voltage Range (V vs. Na^+^/Na)	Specific Capacity (mAh·g^−1^)/Cycles/ Current Density (A·g^−1^)	Rate Performance (mAh·g^−1^)/Current Density (A·g^−1^)	Ref.
CuMn_2_O_4_/ graphene	0.01–3.0	313/50th/0.1	145/2.0	[14]
Fe_2_O_3_-600 nanosheets	0.01–3.0	~100/250th/0.5	100/0.5	[35]
CNF/CoO-4	0.01–3.0	138/100th/0.1	—	[36]
Mn_3_O_4_/rGO aerogels	0.01–3.0	283/100th/0.1	121/1.0	[37]
CoMoO_4_@C	0.5–3.0	46/50th/0.5	45/2.0	[38]
CoMoO_4_ nanorod	0.01–3.0	200/60th/0.1	160/0.5	[39]
ZnMn_2_O_4_/jute porous carbon	0.01–3.0	392.4/200th/0.1	244.7/1.0	[40]
MnV_2_O_6_/GO	0.01–3.0	323.8/100th/0.1	213.8/2.0	[41]
CoMoO_4_@NC	0.01–3.0	281/80th/0.1	221/2.0	[42]
MnFe_2_O_4_@4C	0.01–3.0	250/100th/0.1	182/2.0	This work

## Data Availability

The data presented in this study are available upon request from the corresponding author.

## Supplementary Materials

The following supporting information can be downloaded at: https://www.mdpi.com/article/10.3390/molecules29163912/s1, Figure S1: SEM images of MnFe_2_O_4_ spherical nanoparticles; Figure S2: TGA curves recorded for MnFe_2_O_4_@1C, MnFe_2_O_4_@4C, and MnFe_2_O_4_@8C nanocomposites under Ar flow; Figure S3: CVs at various scan rates and the calculated b values of MnFe_2_O_4_@1C and MnFe_2_O_4_@8C nanocomposite electrodes.
