# Supplementary material for: Investigating the Electrochemical Performance of MnFe2O4@xC Nanocomposites as Anode Materials for Sodium-Ion Batteries
Source: Molecules. 2024 Aug 19;29(16):3912. doi: 10.3390/molecules29163912 (PMC11356791; doi:10.3390/molecules29163912)
Supplement: Supplementary file 1 [file molecules-29-03912-s001.zip › molecules-3105462-supplementary.pdf]

Supplementary Materials

Investigating the Electrochemical Performance of $MnFe_2O_4@xC$ Nanocomposites as Anode Materials for Sodium-Ion Batteries

Shi-Wei Liu [1], Bai-Tong Niu [1], Bi-Li Lin [1], Yuan-Ting Lin [1], Xiao-Ping Chen [1], Hong-Xu Guo [1], Yan-Xin Chen [2], and Xiu-Mei Lin [1,*]

[1] College of Chemistry, Chemical Engineering and Environment, Minnan Normal University, Zhangzhou 363000, China.

[2] State Key Laboratory of Structural Chemistry, Fujian Institute of Research on the Structure of Matter, Chinese Academy of Sciences, Fuzhou 350002, China.

Correspondence: xiu-mei.lin@xmu.edu.cn (X.-M. L.).

Experimental section

Material characterization

XRD patterns were obtained using a Rigaku D/MAX-RB X-ray diffractometer with Cu Kα radiation from Japan. Micro-morphological features were examined via scanning electron microscopy (SEM) using a Regulus 8100 field-emission SEM from Japan and high-resolution transmission electron microscopy (HRTEM) using a Tecnai F30 instrument from the USA. The thermal stability of $MnFe_2O_4$@xC was evaluated using a thermogravimetric analyzer (TGA-4000, Perkin Elmer).

Electrochemical characterization

In this process, the active material ($MnFe_2O_4$@xC), polyvinylidene fluoride (PVDF), and Super P were mixed in a mass ratio of 8:1:1, and then NMP was added to form a slurry. This black slurry was applied onto a Cu foil current collector and vacuum-dried for 12 hours at 120℃. The 2025 stainless steel coin cells were assembled in an Argon-filled glove box (Vigor) with O_2 and H_2O levels maintained below 0.1 ppm. Sodium half-cells were assembled using $MnFe_2O_4$@xC, glass fiber (whatman, GF/C), Na foil, and 1.0 M $NaPF_6$ in DME as the working electrode, separator, counter electrode, and electrolyte, respectively. The battery performance of the assembled cells was assessed utilizing the Neware battery testing system. Cyclic voltammetry (CV) and AC electrochemical impedance spectroscopy (EIS) measurements were conducted using an Autolab electrochemical workstation. The $Na_3V_2(PO_4)_3$ (NVP) cathode was purchased from Suzhou Dodo Chemical Technology Co., Ltd. To assemble the full cell, the cathode electrode material NVP, Super P, and PVDF (in a mass ratio of 8:1:1) were dissolved in NMP, mixed, and then coated onto Al foil followed by drying at 120℃ in a vacuum for 12 hours. The areal mass loading of the anode and cathodes is around 1.5 and 3.0 $mg \cdot cm^{-2}$, respectively. According to the reversible capacity and mass ratio of NVP and $MnFe_2O_4$@4C in the half cells, the N/P ratio was calculated to be 1.2. Prior to the full cell test, the $MnFe_2O_4$@4C anode was pre-sodiumized.

Supplementary Figures

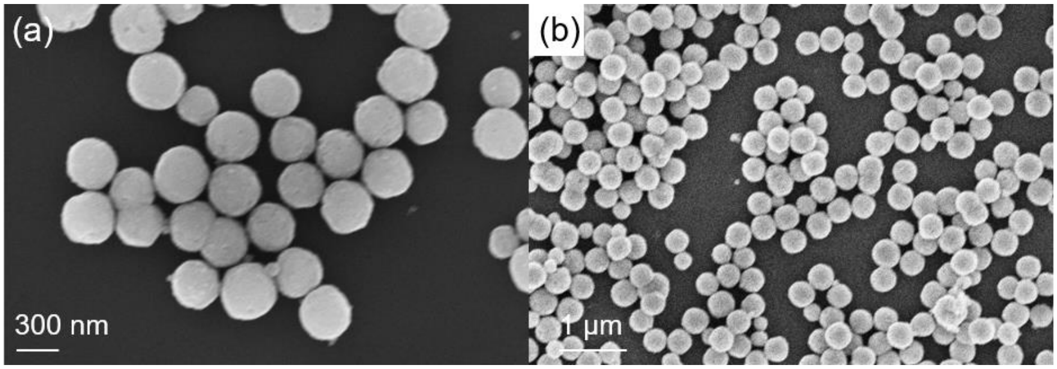

Figure S1. (a, b) SEM images of $MnFe_2O_4$ spherical nanoparticles.

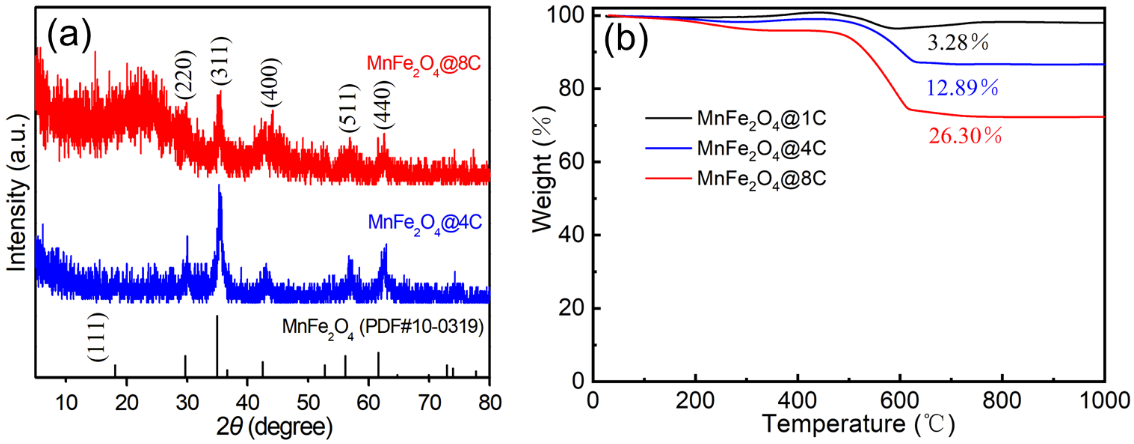

Figure S2. (a) Powder XRD patterns of the $MnFe_2O_4$@4C and $MnFe_2O_4$@8C. (b) TGA curve recorded for $MnFe_2O_4$@1C, $MnFe_2O_4$@4C, and $MnFe_2O_4$@8C nanocomposites under O_2 flow.

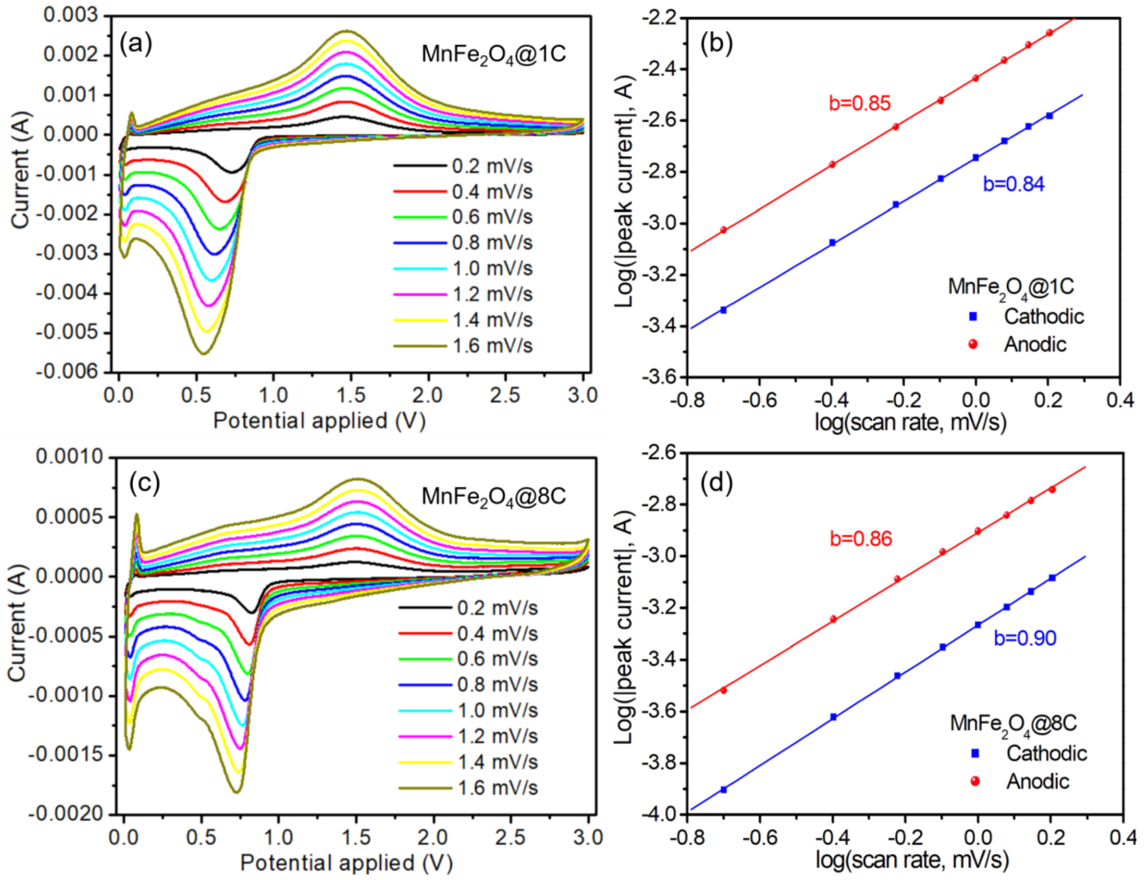

Figure S3. (a, c) CVs at a series of scan rates and (b, d) calculated b value of $MnFe_2O_4$@1C and $MnFe_2O_4$@8C nanocomposite electrodes.
